# Investigation of awareness, sanitation, and customer education practices among employees of pet and animal feed stores that sell live animals in the United States

**DOI:** 10.1186/s12889-024-20881-3

**Published:** 2024-12-05

**Authors:** Jennifer Lord, Sheri Pugh, Sharon R. Thompson

**Affiliations:** 1 Department of Biomedical and Diagnostic Sciences, College of Veterinary Medicine, University of Tennessee, Knoxville, USA; 2 Center for Agriculture and Food Security and Preparedness, Department of Biomedical and Diagnostic Sciences, College of Veterinary Medicine, University of Tennessee, Knoxville, USA; 3 Tennessee Integrated Food Safety Center for Excellence, Knoxville and Nashville, USA

**Keywords:** Zoonotic disease, Public health, Pet store, Animal husbandry, One Health

## Abstract

**Background:**

Numerous zoonotic disease outbreaks have been associated with companion animals and poultry purchased at pet and animal feed stores. Employees are often the initial source of information for customers purchasing a new pet. Therefore, the objectives of this study were to: (1) investigate awareness, sanitation, and customer education practices related to zoonotic disease risk, and (2) identify predictors of providing customer education among employees of pet and animal feed stores that sell live animals.

**Methods:**

A survey of pet and animal feed store employees was conducted to evaluate sanitation practices, training, and awareness of zoonotic disease risk. Differences in proportions of categorical variables were assessed using Chi-square tests. Wilcoxon rank-sum and Kruskal–Wallis tests were used to assess for differences in the values of ordinal variables based on the values of categorical variables. A partial proportional odds model was used to identify predictors of providing customer education.

**Results:**

Surveys were completed by 206 respondents from the Southeast, Midwest, Southwest, and Western US, 146 of whom reported that their workplace sold live animals. Handwashing was more frequent among employees whose workplace had handwashing policies related to handling animals and their habitats (*p* < 0.001). Perceived zoonotic disease risk was higher among those who had received workplace training (*p* = 0.007). Higher odds of providing customer education related to zoonotic disease risk were associated with serving in a supervisory role (*p* = 0.005), higher perceived zoonotic disease risk (*p* = 0.001), and more frequent sanitation practices (handwashing, *p* = 0.031; surface disinfection, *p* < 0.001; and glove use, *p* < 0.001).

**Conclusions:**

Pet and animal feed stores play an important role in minimizing occupational health hazards for employees and providing education for customers. These retailers should implement clear biosecurity protocols and provide training about zoonotic disease risk associated with handling live animals.

**Supplementary Information:**

The online version contains supplementary material available at 10.1186/s12889-024-20881-3.

## Background

Animal ownership can have positive impacts on human health and well-being [1, 2]. More than half of households in the United States have one or more pets [3], and many consider their pets to be a part of the family. However, contact with pets and backyard poultry is associated with an increased risk of exposure to zoonotic pathogens [4–9]. Risk of illness and hospitalization resulting from transmission of zoonotic pathogens is higher among certain groups, including young children (< 5 years old), adults aged 65 and older, people who are pregnant, and those with immune compromise. Transmission can occur through direct contact, including bites and scratches, as well as indirect contact via cross-contamination of objects and surfaces [4, 6, 7, 9].

Bite and scratch wounds from dogs and cats may result in infections such as pasteurellosis and bartonellosis (cat scratch disease) [7, 10, 11]. Animal contact is also an important route of transmission for enteric pathogens; in the US, an estimated 17% of human illnesses due to *Campylobacter* infections and 11% of those due to non-typhoidal *Salmonella* are attributable to animal contact [12]. Numerous species can harbor *Salmonella* in their gastrointestinal tract and shed the organism asymptomatically; however, contact with poultry or non-traditional pets (companion animals other than dogs and cats) such as reptiles and amphibians has repeatedly been associated with zoonotic *Salmonella* transmission [4, 7]. Contact with non-traditional pets can also result in exposure to various other zoonotic pathogens [4]. For example, contact with pet birds infected with *Chlamydia psittaci* is an important cause of psittacosis [13]. Contact with small mammals has been associated with human illness due to *Streptobacillus moniliformis* (the causative agent of rat bite fever) [14–17], hantaviruses [18–20], lymphocytic choriomeningitis virus [21–24], and Mpox [25].

The sale and adoption of animals at retail pet stores and agricultural feed stores may result in the transmission of zoonotic pathogens to employees and customers, and may also facilitate disease spread through the distribution of animals [6]. Shedding of pathogens such as *Salmonella* may be increased due to stress from transport and the combined transportation and housing of animals from different sources [4, 26, 27]. In the US, numerous outbreaks of human illness have been associated with contact with animals purchased at pet and/or feed stores. For instance, a multistate outbreak of extensively drug-resistant *Campylobacter jejuni* infections was attributed to contact with pet store puppies [28]. Contact with young poultry purchased at agricultural feed stores has been implicated in outbreaks of salmonellosis resulting in illness and hospitalization [5, 26, 29–31]. In 2020, record numbers of backyard poultry-associated *Salmonella* outbreaks were detected in the US, with many illnesses occurring among new poultry owners [32]. There have also been numerous multistate outbreaks of human *Salmonella* infection attributable to contact with reptiles or amphibians, primarily affecting infants and young children [33–38].

Previous and ongoing zoonotic disease outbreaks associated with animals purchased at pet and agricultural feed stores highlight the importance of appropriate husbandry and sanitation practices, both within these settings and after purchase [4, 6, 27]. Transmission of zoonotic pathogens often occurs through direct or indirect animal contact at home [4], and findings of previous research suggest that public awareness of zoonotic disease risk is relatively low [30, 33, 36, 37, 39, 40]. Employees of pet and animal feed stores where companion animals and live poultry are sold often serve as the initial source of information for customers purchasing a new pet [4, 6]. However, there is limited published information on awareness of zoonotic disease risk and customer education practices among employees of pet and animal feed stores that sell live animals in the United States. Given the important role of these retailers with respect to customer education about zoonotic disease risk, understanding awareness and current workplace practices is crucial [27]. Identifying factors associated with providing customer education can guide the development of evidence-based outreach strategies to promote this practice and prevent zoonotic disease transmission associated with pets and backyard poultry. Therefore, the objectives of this study were to: (1) investigate awareness, sanitation, and customer education practices related to zoonotic disease risk, and (2) identify predictors of providing customer education among employees of pet and animal feed stores that sell or offer onsite adoption of live animals.

## Materials and methods

### Ethics approval and consent to participate

This study was performed in accordance with the Declaration of Helsinki and was reviewed and approved as exempt by the University of Tennessee Institutional Review Board Human Research Protections Program under 45 CFR 46.101 (Number: UTK IRB-23-07792-XM). Participation in the survey was voluntary, and all results are presented in aggregated form with no identifying information to ensure that participants cannot be re-identified. Since the study was classified as secondary research and investigators had no additional contact with and did not attempt to re-identify study subjects, additional informed consent was not required by the Institutional Review Board.

### Survey development and dissemination

A survey of pet and animal feed store employees was developed and conducted by the University of Tennessee Center for Agriculture and Food Security and Preparedness (CAFSP), part of the Tennessee Integrated Food Safety Center of Excellence. The survey, which was designed to evaluate sanitation practices, training, and awareness of zoonotic disease risk related to animal husbandry and food handling to assess the need for development of educational materials, was created and implemented using QuestionPro Research Edition software. The survey was developed with input from subject matter experts and reviewed by public health experts from the Tennessee Department of Health (TDH) and Centers for Disease Control and Prevention (CDC). Survey parameters were identified based upon input from these agencies. The survey was revised numerous times as part of this review process prior to distribution.

Surveys were distributed to pet and animal feed stores across the US via email. Flyers containing a quick-response (QR) code were also distributed to store managers for display. Employees could access the survey by scanning the QR code with a smartphone. A voluntary gift card drawing was added as an incentive for completing the survey and managers were contacted by phone to solicit participation. In total, 176 respondents started the original survey, but only 16 (9.1%) of these were completed. Feedback from managers and employees indicated that the low completion rate was due to the length of the survey, which was subsequently revised and re-distributed. This revised survey consisted of 33 questions in multiple-choice (single and multiple response), 5-point Likert-type scale (always to never), and 7-point Likert-type scale (strongly agree to strongly disagree) formats. In total, survey development, review and revision resulted in the construction of at least five drafts. The revised survey was used to collect the data reported in the current study. The survey has not been previously published and is included as Additional File 1.

Store managers were contacted by phone and via email to seek participation in the revised survey. With store manager permission, paper survey packets were hand-delivered or mailed to pet and animal feed stores. Surveys were distributed to stores in Alabama, Arizona, California, Colorado, Florida, Georgia, Iowa, Indiana, Illinois, Kansas, Kentucky, Massachusetts, Minnesota, North Carolina, Ohio, Oregon, Pennsylvania, Tennessee, Texas, Washington, and Wisconsin. Existing CAFSP contractors assisted with identification of pet and animal feed stores and dissemination of survey packets in their area. Student workers at other Centers of Excellence also assisted with survey distribution.

Store employees received three options for accessing the survey: 1) scanning a QR code, 2) typing an address into their web browser, or 3) completing a paper copy. For those who elected to use a paper copy, postage-paid envelopes were provided for returning completed surveys. Movie theater candy was provided as an incentive for completing the survey in addition to the gift card drawing. Surveys were completed between July 2021 and October 2021.

### Data management and descriptive statistics

Data management, visualization, and statistical analysis were performed using R version 4.4.0 [41]. Survey respondents who answered “Yes” to the question “Does your current workplace(s) sell live animals or offer onsite adoption of live animals?” were included in the current study. Ordinal variables were coded such that higher values indicated either more frequently performing a task (1 = Never, 2 = Rarely, 3 = Sometimes, 4 = Often, 5 = Always) or stronger agreement with a statement (1 = Strongly disagree, 7 = Strongly agree).

Chi-square tests were used to assess for significant differences in proportions of categorical variables. Wilcoxon rank-sum tests were used to assess for significant differences in the values of ordinal variables based on the values of dichotomous variables, and Kruskal–Wallis tests were used to assess for significant differences in the values of ordinal variables based on the values of non-ordered categorical variables with three or more categories. A *p*-value < 0.05 was considered statistically significant.

### Investigation of predictors of customer education practices

Cumulative logit models were used to identify characteristics associated with how often respondents provided customer education related to zoonotic disease risk [42, 43]. These models relate the probability of a participant’s response being at or below a given category to the probability of being in a higher category. The outcome was an ordinal variable with response categories ranging from 1 (Never) to 5 (Always). Models were fit using the clm function from the ‘ordinal’ package in R [44].

To prevent problems with multicollinearity, correlations between all possible pairs of potential predictors were assessed using Spearman rank-order correlation tests. If a pair of variables was highly correlated (|*r*_*s*_|> 0.7), one was selected for consideration in the model-building process. Univariable logistic regression models were used to assess associations between pairs of dichotomous predictor variables. Only one of a pair of variables with an odds ratio (OR) > 8.0 was considered for inclusion in the model-building process.

Model-building was performed in two steps. First, univariable associations between each potential predictor variable and the outcome were assessed using constrained cumulative logit models (proportional odds models) to identify candidate variables for inclusion in the multivariable model. Statistical significance was assessed using likelihood ratio tests. Variables associated with the outcome at a liberal *p*-value of < 0.20 were considered for inclusion in the multivariable model. In addition, workplace type and working at a store that sold live poultry were considered in the multivariable modeling process despite having *p*-values > 0.2 for the univariable association. These variables were considered due to hypothesized associations with the outcome and to permit investigation of these variables as potential confounders. In the second step, a multivariable proportional odds model was built using manual backwards elimination, specifying a critical *p*-value of 0.05 for inclusion in the final model. If the removal of a variable changed the magnitude of the coefficient of another variable by > 20%, that variable would be considered a potential confounder and retained in the final model regardless of statistical significance. Variance inflation factors (VIFs) were computed to assess for multicollinearity, with VIFs > 10 indicating serious problems with multicollinearity.

The proportional odds assumption was assessed for each variable in the model using the Brant test [45]. When the proportional odds assumption was not met for all variables in the model, a partial proportional odds model was fit. This model allows the coefficients for predictors that did not meet the proportional odds assumption to vary across levels of the outcome. An approximate likelihood ratio test was used to assess whether the partial proportional odds model had better fit to the data than the proportional odds model.

## Results

### Descriptive statistics

Among the 221 respondents who started surveys, 206 were completed. A total of 146 (70.9%) respondents indicated that their current workplace sold and/or offered onsite adoption of live animals and were included in subsequent analyses. Approximately half (51.8%) of these respondents reported working at a regional or national chain, while 29.1% worked at a local chain and 19.1% worked at an independent store (Table 1). The most common types of animals sold or offered for adoption included chickens and other poultry (61.6%), small mammals (guinea pigs, hamsters, rodents, and rabbits, 56.8%), and reptiles and amphibians (30.1%).

**Table 1:** Workplace characteristics and activities reported by employees of U.S. pet and animal feed stores that sell live animals

Characteristic	Number	Percent
*Type of workplace*		
National or regional chain	73	51.8%
Local chain	41	29.1%
Independent store	27	19.1%
*Type of animals sold*		
Chickens and other poultry	90	61.6%
Small mammals (guinea pigs, hamsters, rodents, rabbits)	83	56.8%
Reptiles and amphibians	44	30.1%
Pet birds (parakeets, parrots, among others)	35	24.0%
Ferrets	28	19.2%
Cats	27	18.5%
Dogs	13	8.9%
*Workplace activities*		
Stocking	117	80.1%
Cashier	115	78.8%
Sales	113	77.4%
Animal care or grooming	86	58.9%
Cleaning of animal housing	70	47.9%
Supervision of one or more employees	46	31.5%
Workplace training on zoonotic disease risk associated with handling live animals	96	65.8%
*Workplace handwashing policies*		
Handling live animals	87	59.6%
Handling animal habitats	68	46.6%
*Workplace educational materials about zoonotic disease risk*		
Handouts provided to customers	62	42.5%
Posters/signs displayed	41	28.1%

The most common workplace activities among respondents included stocking (80.1%), cashier (78.8%), and sales (77.4%). A total of 58.9% of respondents performed animal care or grooming, and 47.9% cleaned animal housing (e.g. pens, cages, food bowls, and water bowls). Just under two-thirds (65.8%) reported that their workplace had provided training on the risk of zoonotic disease associated with handling live animals. A total of 59.6% reported that their workplace had specific handwashing policies related to handling live animals, and 46.6% had specific handwashing policies related to handling animal habitats. While 42.5% of respondents reported that their workplace provided handouts or other take-home information to customers on the risk of zoonotic disease associated with handling live animals, fewer (28.1%) had posters or other signage displayed.

**Fig. 1:**
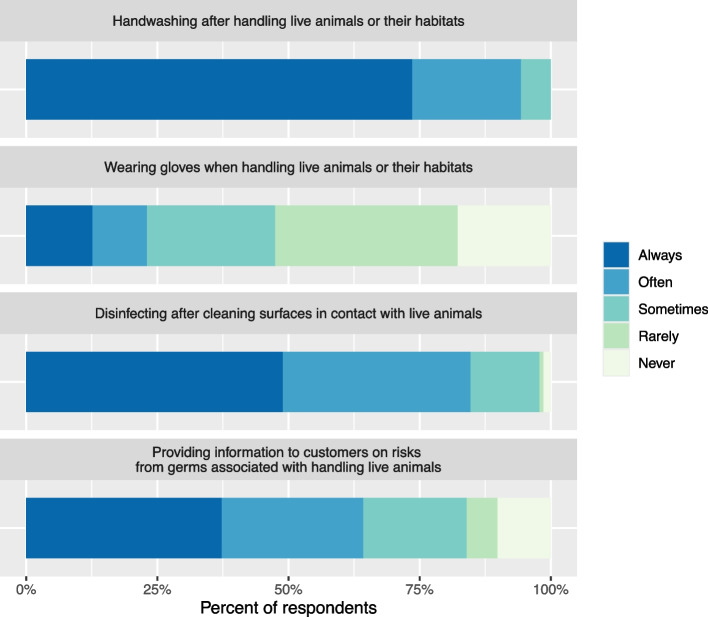
Frequency of sanitation and customer education practices among employees of U.S. pet and animal feed stores that sell live animals

The majority of respondents (73.6%) reported that they always washed their hands after handling live animals or their habitats, while 20.7% washed their hands often and 5.7% sometimes washed their hands (Fig. 1). The use of gloves when handling animals or their habitats was less frequent; just 12.6% of respondents always wore gloves, while approximately half (52.6%) either rarely or never wore gloves. Most respondents either always (48.9%) or often (35.8%) used a disinfectant after cleaning surfaces in contact with live animals. A total of 37.2% of respondents reported that they always provided information to customers on zoonotic disease risk associated with handling live animals, and 27.0% often provided this information. However, 5.8% and 10.2% of respondents rarely or never provided this information to customers, respectively. The majority (60.4%) strongly agreed that people can become ill from touching or handling live animals, including pets or poultry.

No significant differences in the frequency of sanitation practices (handwashing: χ^2^ = 0.94, *p* = 0.6238; surface disinfection: χ^2^ = 2.28, *p* = 0.3197; glove use: χ^2^ = 1.15, *p* = 0.5621) or customer education (χ^2^ = 2.78, *p* = 0.2478) were identified based on workplace type. A total of 72.6% of those who worked at national or regional chains had received zoonotic disease training, compared to 61.0% of employees of local chains and 66.7% of employees of independent stores, but this difference was not significantly different (${\chi }^{2}$ = 1.66, *p* = 0.4351). Handwashing after handling animals and their habitats was significantly more frequent among employees whose workplace had specific handwashing policies related to handling live animals (*W* = 1607.5, *p* < 0.0001) and animal habitats (*W* = 1734, *p* = 0.0001).

There was no significant difference in perceived zoonotic disease risk (χ^2^ = 0.1451, *p* = 0.9300) based on workplace type. However, perceived zoonotic disease risk was significantly (*W* = 1741, *p* = 0.0068) higher among respondents that had received workplace training on the risk of zoonotic disease associated with handling live animals compared to those who had not. There was no significant difference in the frequency of handwashing after handling animals and their habitats based on whether a respondent had received zoonotic disease training at their workplace (*W* = 1796, *p* = 0.0659). However, the frequency of surface disinfection (*W* = 1403.5, *p* = 0.0037) and glove use (*W* = 1272.5, *p* = 0.0006) were significantly higher among those who had received such training. Respondents that received workplace training on zoonotic disease risk also provided information on zoonotic disease risk to customers significantly more frequently (*W* = 1077.5, *p* < 0.0001) than those who did not.

### Investigation of predictors of customer education practices

Univariable associations between workplace and employee characteristics and the frequency of providing customer education on zoonotic disease risk are displayed in Table 2. Sanitation practices (*p* < 0.0001), perceived zoonotic disease risk (*p* = 0.0017), workplace zoonotic disease training (*p* < 0.0001), and handwashing policies related to handling live animals (*p* = 0.0051) and their habitats (*p* = 0.0157) had significant univariable associations with the outcome. Of note, working at a store that sold reptiles was associated with higher odds of performing animal care or grooming activities (Odds Ratio [OR] = 12.2, *p* < 0.0001). In addition, respondents whose workplace had specific handwashing policies related to handling live animals also had significantly higher odds of having specific handwashing policies related to handling animal habitats (OR = 38.3, *p* < 0.0001).

**Table 2:** Univariable associations between workplace or employee characteristics and customer education practices among employees of U.S. pet and animal feed stores that sell live animals

**Variable**	**OR**^a^	**95% CI**^b^		***p*-value (LRT**^c^**)**	***p*-value** **(Brant test)**
*Type of workplace*					
Regional or national chain	Ref	-	-	0.2351	-
Local chain	0.53	0.26	1.10	0.0889	0.16
Independent store	0.81	0.36	1.81	0.6024	0.46
*Type of animals sold*					
Dogs	0.62	0.20	1.93	0.4020	0.01
Cats	0.83	0.38	1.79	0.6250	0.05
Ferrets	1.30	0.62	2.73	0.4855	0.10
Guinea pigs, hamsters, rodents, rabbits	1.40	0.76	2.60	0.2791	0.12
Reptiles & amphibians	1.57	0.81	3.08	0.1787	0.48
Chickens & other poultry	0.83	0.45	1.55	0.5664	0.94
Pet birds	1.30	0.65	2.64	0.4559	0.19
*Workplace activity*					
Sales	1.28	0.60	2.70	0.5239	0.34
Cashier	1.36	0.62	2.96	0.4349	0.74
Animal care or grooming	1.63	0.86	3.07	0.1322	0.59
Stocking	0.73	0.30	1.72	0.4815	0.38
Supervision	1.76	0.92	3.40	0.0861	0.84
Cleaning animal housing	0.33	0.72	2.44	0.3630	0.31
*Sanitation practices*					
Handwashing after handling live animals or habitats	3.32	1.94	5.79	< 0.0001	0.61
Wearing gloves when handling live animals or habitats	1.81	1.39	2.39	< 0.0001	0.17
Surface disinfection after cleaning	2.59	1.75	3.90	< 0.0001	0.20
Perceived zoonotic disease risk	1.43	1.14	1.79	0.0017	0.71
Zoonotic disease training	5.04	2.52	10.3	< 0.0001	0.57
*Workplace handwashing policies*					
For handling live animals	2.46	1.31	4.68	0.0051	0.54
For handling animal habitats	2.13	1.15	3.96	0.0157	0.26

^a^Odds ratio

^b^Confidence interval

^c^Likelihood ratio test

Four of the five variables in the final multivariable model met the proportional odds assumption: supervising one or more other employees, perceived zoonotic disease risk associated with handling live animals, handwashing after handling animals or their habitats, and disinfecting after cleaning surfaces in contact with animals (Table 3). The Brant test indicated a violation of the proportional odds assumption for the frequency of glove use when handling live animals or their habitats (*p* < 0.0001). Therefore, a partial proportional odds model was fit to the data. Workplace zoonotic disease training was a significant predictor in the proportional odds model (*p* = 0.0466). However, after fitting the partial proportional odds model to the data to account for the non-proportional odds of the glove use variable, the association between zoonotic disease training and frequency of providing customer education was no longer statistically significant (*p* = 0.0730). In addition, the value of Akaike’s information criterion (AIC) for the full model (AIC = 328.12) was not substantially improved compared to the reduced model (AIC = 329.34). Therefore, the zoonotic disease training variable was removed and the more parsimonious model selected as the final model. Examination of the percent change in coefficients after variable elimination during the model-building process did not reveal evidence of confounding.

**Table 3:** Multivariable partial proportional odds model predicting customer education practices among employees of U.S. pet and animal feed stores that sell live animals

	**OR**^a^	**95% CI**^b^		***p*-value**
*Proportional odds*				
Supervises other employees	2.80	1.36	5.92	0.0050
Perceived zoonotic disease risk	1.51	1.18	1.95	0.0013
Handwashing after handling live animals or their habitats	1.91	1.06	3.49	0.0308
Disinfecting after cleaning surfaces in contact with animals	2.46	1.60	3.84	< 0.0001
*Non-proportional odds*				
Wearing gloves when handling animals or their habitats				< 0.0001
Rarely, sometimes, often, or always (vs. never)	1.54	0.36	6.53	0.5581
Sometimes, often, or always (vs. rarely or never)	4.71	1.99	11.1	0.0004
Often or always (vs. sometimes, rarely, or never)	2.15	1.45	3.21	0.0002
Always (vs. often, sometimes, rarely, or never)	1.52	1.10	2.10	0.0110

^a^Odds ratio

^b^Confidence interval

Respondents who supervised one or more other employees at their workplace had significantly higher odds of providing customers with zoonotic disease information compared to those who did not have a supervisory role. For respondents with a supervisory role, the odds of providing customer education more frequently were 2.8 times that of those without a supervisory role (*p* = 0.0050). In addition, those with higher perceived zoonotic disease risk also provided information to customers more frequently. A unit increase in perceived zoonotic disease risk was associated with 1.51 times higher odds of more frequent customer education (*p* = 0.0013). More frequent adherence to sanitation practices was also associated with the frequency of customer education. Higher frequency of handwashing after handling live animals (OR = 1.91, *p* = 0.0308) and surface disinfection (OR = 2.46, *p* < 0.0001) were associated with higher odds of providing customers with zoonotic disease information. Respondents who used gloves more frequently also tended to have higher odds of providing information on zoonotic disease risk to customers, but these odds differed based on the response level of the outcome (Table 3). For instance, a unit increase in the frequency of glove use was associated with 4.71 times higher odds of providing customer education sometimes, often, or always (compared to rarely or never providing this information). However, a unit increase in the frequency of glove use was associated with 2.15 times higher odds of providing customer education often or always (compared to sometimes, rarely, or never).

## Discussion

Contact with household pets and backyard poultry is a potential route of transmission for zoonotic pathogens that cause preventable illness in humans. Employees of pet and animal feed stores, who are at risk of occupational exposure to zoonotic pathogens, often serve as the initial source of husbandry and hygiene information for customers purchasing a new pet [4, 6]. Therefore, this study investigated awareness, sanitation, and customer education practices related to zoonotic disease risk among employees of pet and animal feed stores in the US that sell or offer onsite adoption of live animals.

Handwashing and surface disinfection were common practices among employees of pet and animal feed stores, with 73.6% reporting that they always washed their hands after handling animals or their environments, and 48.9% reporting that they always used disinfectant after cleaning surfaces in contact with live animals. However, glove use was much less frequent, and over half (52.6%) of respondents either rarely or never wore gloves while handling live animals or their habitats. The reason for this finding was unclear, although frequency of glove use could be impacted by factors such as employee preferences and availability of personal protective equipment. A previous survey of agricultural feed stores in Pennsylvania, conducted as part of an outbreak investigation of human *Salmonella* infection associated with contact with live poultry, reported that just 20% offered personal protective equipment to employees who handled live birds or cleaned their environments [30]. Findings of prior research suggest that the use of personal protective equipment when handling live animals or cleaning their habitats is also uncommon among members of the public [40]. Wearing gloves can reduce exposure to zoonotic pathogens when combined with effective handwashing after their removal. The use of thicker work gloves may also be appropriate in certain situations to prevent injuries due to bites and scratches, which can result in transmission of pathogens such as *Streptobacillus moniliformis* [14].

Written biosecurity protocols, including handwashing policies, are recommended for employees of pet and agricultural retail stores to prevent the transmission of zoonotic pathogens from live poultry and pets to people [4, 27]. The aforementioned Pennsylvania survey reported that only 20% of feed stores had a written handwashing policy for employees [30]. In the current study, however, 59.6% of respondents reported that their workplace had handwashing policies related to handling live animals, and 46.6% had handwashing policies related to handling animal habitats. Handwashing after handling animals and their environments in the workplace was significantly more frequent among those who reported that these policies were in place. This finding provides evidence in support of implementing handwashing policies as a strategy to promote adherence to recommended hygiene practices. Findings of this study also support recommendations for the provision of workplace training on zoonotic disease risk to improve employees’ awareness and sanitation practices [4, 27]. Perceived disease risk and the frequency of surface disinfection, glove use, and customer education were all significantly higher among respondents who had received zoonotic disease training compared to those who had not.

The majority of respondents agreed (11.8%) or strongly agreed (60.4%) with the statement that people can become ill from touching or handling live animals, including pets or poultry, suggesting that most retail employees are aware of zoonotic disease risk at their workplace. This is consistent with findings of previous research that investigated awareness of *Salmonella* risk associated with live poultry among representatives of agricultural feed stores in Pennsylvania (74%) and New Mexico (85%) [26, 30]. However, findings of those studies indicated a much lower percentage of store representatives (28% of those from Pennsylvania and 57% from New Mexico) provided this information to customers [26, 30]. This is also in line with findings of the current study, which suggest that customer education on zoonotic disease prevention does not occur consistently at pet and animal feed stores in the US. Numerous expert groups have recommended the display and delivery of educational materials related to zoonotic disease risk in multiple formats and languages by live animal retailers [4–6, 9, 27]. However, fewer than half (42.5%) of respondents in this study reported that their workplace provided take-home information to customers on the risk of zoonotic disease associated with handling live animals, and just 28.1% had posters or other signage displayed.

Although most respondents strongly agreed that contact with live animals was associated with zoonotic disease risk, a far lower percentage (37.2%) reported that they always provided zoonotic disease awareness education to customers. Furthermore, some employees (16%) reported that they rarely or never provided this information, suggesting that there is room for improvement with respect to customer education in pet and animal feed stores. Indeed, findings of previous research suggest that public awareness of zoonotic disease risk associated with household pets, including reptiles, amphibians, and backyard poultry, is relatively low [30, 33, 36, 37, 39, 40].

Given the important role of pet and animal feed retailers with respect to public education about zoonotic disease risk, this study sought to identify predictors of how frequently survey respondents provided customers with this information. Those who served in a supervisory role at their workplace had significantly higher odds of providing customer education on zoonotic disease risk. These employees may be more experienced and knowledgeable, and may also be more comfortable interacting and communicating with customers. In addition, employees with higher perceived zoonotic disease risk and those who adhered to recommended sanitation practices more frequently had higher odds of providing customer education. Workplace zoonotic disease training was not a significant predictor of customer education practices in the final model. This finding may be attributable to associations between zoonotic disease training and other variables in the model (perceived zoonotic disease risk and sanitation practices). Interestingly, while contact with certain animals (e.g. reptiles, amphibians, poultry) is associated with relatively greater risk of transmission of zoonotic pathogens than others [4, 7], type of animal(s) sold was not associated with customer education practices. Similarly, type of animal(s) sold was not significantly associated with frequency of adherence to sanitation practices or perceived zoonotic disease risk.

Effective employee and customer education about zoonotic disease risk and appropriate pet selection, husbandry, and hygiene practices have the potential to decrease transmission of zoonotic pathogens and prevent illness due to contact with live animals and their habitats [4–6, 26, 27, 29–31, 34, 35, 37]. Findings of this study support the inclusion of specific handwashing policies in written biosecurity protocols for employees to promote adherence to recommended hygiene practices. Further research is warranted to assess whether the limited glove use among pet and animal feed store employees observed in this study is attributable to availability of personal protective equipment or other factors. Findings of this study also suggest that the display of educational signage and provision of take-home materials related to zoonotic disease risk does not occur consistently in pet and animal feed stores in the US. This study also found that employees with more experience, awareness and more frequent adherence to recommended sanitation practices in the workplace had higher odds of providing customer education on zoonotic disease risk. Therefore, increasing knowledge and awareness of employees who work in these settings may translate to improved customer education, which has the potential to improve safe hygiene and handling practices beyond the store setting. Implementing regular employee zoonotic disease training, which was associated with higher perceived zoonotic disease risk and more frequent sanitation practices, represents an important strategy to improve awareness and workplace safety.

### Strengths and limitations

This study provides useful information on awareness, sanitation and customer education practices among employees of live animal retailers in the United States. Respondents from a wide geographic range participated in the survey, representing every region except for the Northeastern US. However, there are a few limitations worth noting. Participation was voluntary, and results may not be generalizable to all employees of pet and animal feed stores that sell live animals in the US if characteristics of survey respondents differed from those who did not respond to the survey. In addition, recall and social desirability biases may have affected participants’ responses to the survey [46]. Despite the above limitations, findings of this study are useful to guide outreach strategies aimed at improving awareness and reducing exposure to zoonotic pathogens associated with animal contact in pet and animal feed stores.

## Conclusions

Commercial live animal retailers play an important role in minimizing occupational health hazards for employees and providing educational resources for customers. Findings of this study highlight gaps in workplace training, policies, and customer education practices among employees of pet and animal feed stores that sell or offer adoption of live animals in the US. Study findings indicate that employees with higher perceived disease risk and who adhere to recommended sanitation practices more frequently are more likely to provide customer education. Pet and animal feed stores should implement clear and specific biosecurity protocols and provide employee training about zoonotic disease risk associated with handling the animal species sold at their workplace.

## Supplementary Information

Supplementary Material 1.

## Data Availability

The data that support the findings of this study are available from the corresponding author upon reasonable request.

## Abbreviations

CAFSP: Center for Agriculture and Food Security and Preparedness
CDC: Centers for Disease Control and Prevention
CI: Confidence Interval
OR: Odds Ratio
QR: Quick-Response
TDH: Tennessee Department of Health
US: United States
