# Supplementary material for: Investigation of awareness, sanitation, and customer education practices among employees of pet and animal feed stores that sell live animals in the United States
Source: BMC Public Health. 2024 Dec 5;24:3382. doi: 10.1186/s12889-024-20881-3 (PMC11619681; doi:10.1186/s12889-024-20881-3)
Supplement: Supplementary file 1 — Supplementary Material 1. [file 12889_2024_20881_MOESM1_ESM.docx]

| **University of Tennessee Pet Store/Feed Store Survey**  The University of Tennessee is conducting this survey to examine and evaluate current practices being applied in pet and feed stores regarding the handling of animals and animal food in these stores. We are collecting this information to assess the need for development of educational materials to assist stores and employees to effectively address risks associated with animals and animal food. We appreciate your participation in this survey and value your honest input. Please note that your responses will be kept confidential and that survey results will only be reported in aggregate form and no identifier information will be released. We are giving away four $25 gift cards to four randomly selected participants that complete the survey. Contact information will only be used to contact the winners of the gift cards.  Please start the survey now by clicking on the **Start** button below.  **LIVE ANIMAL SECTION**  The following questions relate to selling live animals or offering onsite adoption of live animals.   1. Does your current workplace(s) sell live animals or offer onsite adoption of live animals?   Yes  (Please continue with Question #2 in this **BLUE Section**)  No (Please jump to Question #10 in the **GREEN Section** below)   1. Choose all of the animals that your current workplace sells or sponsors during onsite adoptions.   Dogs  Cats  Ferrets  Guinea Pigs, Hamsters, Rodents, Rabbits  Reptiles and Amphibians (e.g. frogs, etc.)  Chickens and other Poultry  Pet Birds such as Parakeet, Parrots, etc.  Other (Please list any other animals that your workplace sells or sponsors on site adoption of that were not included above.)   \|  \| \| --- \|   3. Do you wash your hands after handling animals or their habitats?   \| Never  1 \| Rarely  2 \| Sometimes  3 \| Often  4 \| Always  5 \| Not Applicable \| \| --- \| --- \| --- \| --- \| --- \| --- \| \|  \|  \|  \|  \|  \|  \|   4. Do you wear gloves when handling animals or their habitats?   \| Never  1 \| Rarely  2 \| Sometimes  3 \| Often  4 \| Always  5 \| Not Applicable \| \| --- \| --- \| --- \| --- \| --- \| --- \| \|  \|  \|  \|  \|  \|  \|   5. Do you routinely use a disinfectant after cleaning surfaces that have been in contact with live animals?   \| Never  1 \| Rarely  2 \| Sometimes  3 \| Often  4 \| Always  5 \| Not Applicable \| \| --- \| --- \| --- \| --- \| --- \| --- \| \|  \|  \|  \|  \|  \|  \|   6. Do you provide information to customers on risks from germs associated with handling of live animals?   \| Never  1 \| Rarely  2 \| Sometimes  3 \| Often  4 \| Always  5 \| Not Applicable \| \| --- \| --- \| --- \| --- \| --- \| --- \| \|  \|  \|  \|  \|  \|  \|   This question relates to your opinion or knowledge of certain topics.  7. People can become ill from touching or handling live animals, including pets or poultry.   \| Strongly Disagree  1 \| 2 \| 3 \| 4 \| 5 \| 6 \| Strongly Agree  7 \| Not Applicable \| \| --- \| --- \| --- \| --- \| --- \| --- \| --- \| --- \| \|  \|  \|  \|  \|  \|  \|  \|  \|   8. We want to gather basic information on use of handwashing policies for employees. Choose all that apply to your current workplace’s handwashing policies for employees. My workplace has _____.  General handwashing policies for employees (e.g. requiring employees to wash their hands after using the restroom, before work and after work, after breaks, etc.)  Handwashing policies for employees when handling live animals.  Handwashing policies for employees when handling animal habitats.  9. Choose all that apply to your current workplace:  Posters/signs are displayed about germs people could get from live animals.  Handouts or other types of take-home information are provided for customers about germs that people could get from live animals. |
| --- | --- | --- | --- | --- | --- | --- | --- | --- | --- | --- | --- | --- | --- | --- | --- | --- | --- | --- | --- | --- | --- | --- | --- | --- | --- | --- | --- | --- | --- | --- | --- | --- | --- | --- | --- | --- | --- | --- | --- | --- | --- | --- | --- | --- | --- | --- | --- | --- | --- | --- | --- | --- | --- | --- | --- | --- | --- | --- | --- | --- | --- | --- | --- | --- | --- |

| **PET FOOD, POULTRY FEED, LIVESTOCK FEED SECTION**  The following questions relate to stores that sell animal food.   1. Does your current workplace(s) sell pet food, poultry feed, or livestock feed?   Yes (Please continue with Question #11 in this **GREEN Section**)  No  (Please jump to Question #19 in the **ORANGE Section** below)   1. Choose all that apply for your current workplace. My workplace:   Sells animal food or pet treats in bags.  Sells animal food or pet treats in bulk.  Sells raw pet food for dogs and/or cats.  Sells fresh, frozen, or live feeder animals such as pinkie mice, rats, or quail.  Mixes animal food for poultry and livestock (e.g. animal food mixing consists of taking ingredients such as soybean meal, ground corn, vitamins and minerals and mixing them into a ration).   1. Do you wear gloves when handling animal food or pet treats?  \| Never  1 \| Rarely  2 \| Sometimes  3 \| Often  4 \| Always  5 \| Not Applicable \| \| --- \| --- \| --- \| --- \| --- \| --- \| \|  \|  \|  \|  \|  \|  \|  1. Do you use a disinfectant after cleaning surfaces that have been in contact with animal food or pet treats?      \| Never  1 \| Rarely  2 \| Sometimes  3 \| Often  4 \| Always  5 \| Not Applicable \| \| --- \| --- \| --- \| --- \| --- \| --- \| \|  \|  \|  \|  \|  \|  \|  1. Do you provide information to customers on risks from germs associated with handling of animal food or pet treats?  \| Never  1 \| Rarely  2 \| Sometimes  3 \| Often  4 \| Always  5 \| Not Applicable \| \| --- \| --- \| --- \| --- \| --- \| --- \| \|  \|  \|  \|  \|  \|  \|  1. Do you wash your hands after handling animal food or pet treats?  \| Never  1 \| Rarely  2 \| Sometimes  3 \| Often  4 \| Always  5 \| Not Applicable \| \| --- \| --- \| --- \| --- \| --- \| --- \| \|  \|  \|  \|  \|  \|  \|   The following question relates to your opinion or knowledge of certain topics.   1. People can become ill after touching or handling animal food or pet treats?      \| Strongly Disagree  1 \| 2 \| 3 \| 4 \| 5 \| 6 \| Strongly Agree  7 \| Not Applicable \| \| --- \| --- \| --- \| --- \| --- \| --- \| --- \| --- \| \|  \|  \|  \|  \|  \|  \|  \|  \|  1. We want to gather basic information on use of handwashing policies for employees. Choose all that apply to your current workplace’s handwashing policies for employees. My workplace has _____.   General handwashing policies for employees (e.g. requiring employees to wash their hands after using the restroom, before work and after work, after breaks, etc.)  Handwashing policies for employees when handling fresh, frozen, or live feeder animals such as pinkie mice, rats, or quail  Handwashing policies for employees when handling animal food or pet treats   1. Choose all that apply to your current workplace:   Posters/ signs are displayed about germs people could get from handling animal food or pet treats.  Handouts or other types of take-home information are provided to customers about germs that people could get from handling animal food or pet treats. |
| --- | --- | --- | --- | --- | --- | --- | --- | --- | --- | --- | --- | --- | --- | --- | --- | --- | --- | --- | --- | --- | --- | --- | --- | --- | --- | --- | --- | --- | --- | --- | --- | --- | --- | --- | --- | --- | --- | --- | --- | --- | --- | --- | --- | --- | --- | --- | --- | --- | --- | --- | --- | --- | --- | --- | --- | --- | --- | --- | --- | --- | --- | --- | --- | --- |

| **WORKPLACE PRACTICES AND TRAINING**  The following set of questions relate to practices in your current workplace.   1. Handwashing materials such as soap and water are readily available.  \| Strongly Disagree  1 \| 2 \| 3 \| 4 \| 5 \| 6 \| Strongly Agree  7 \| Not Applicable \| \| --- \| --- \| --- \| --- \| --- \| --- \| --- \| --- \| \|  \|  \|  \|  \|  \|  \|  \|  \|  1. Do you routinely use an alcohol-based sanitizer for hand hygiene in your current workplace?  \| Never  1 \| Rarely  2 \| Sometimes  3 \| Often  4 \| Always  5 \| Not Applicable \| \| --- \| --- \| --- \| --- \| --- \| --- \| \|  \|  \|  \|  \|  \|  \|  1. What is the minimum length of time needed for hand sanitizer to kill most germs on your hands? Select one answer only.   3 seconds  10 seconds  20 seconds  1 minute  I don’t know   1. What is the minimum length of time recommended for handwashing to remove most germs from your hands? Select one answer only.   3 second  10 seconds  20 second  1 minute  I don’t know  The following set of questions relate to training provided in your workplace.   1. Have you received training from your workplace in ways to wash and sanitize your hands in the last three years?   Yes  No  Don’t know/not sure  Not applicable   1. In what format did you receive the training?   Handout materials  In-person training  Online training  Other. If Other, please describe the format of the training.   \|  \| \| --- \|  1. Has your workplace provided training to you about the potential risk of germs spread to people from any of the following? (Choose all that apply)   From live animals to people  From handling animal food or pet treats  From handling feeder animals (i.e. live, fresh or frozen rodents, or other species that are fed to other animals)   1. In what format did you receive the training?   Handout materials  In-person training  Online training  Other. If Other, please describe the format of the training.   \|  \| \| --- \|   The following set of questions relates to training/information needs you may have. Please indicate how much you agree with the following statements.   1. More information/training on handwashing and cleaning and disinfection procedures when handling animals, their habitats or animal food would be helpful to me.  \| Strongly Disagree  1 \| 2 \| 3 \| 4 \| 5 \| 6 \| Strongly Agree  7 \| Not Applicable \| \| --- \| --- \| --- \| --- \| --- \| --- \| --- \| --- \| \|  \|  \|  \|  \|  \|  \|  \|  \|  1. More information/training on germs that people can get from contact with or by handling live animals, their habitats, or animal food would be helpful to me.  \| Strongly Disagree  1 \| 2 \| 3 \| 4 \| 5 \| 6 \| Strongly Agree  7 \| Not Applicable \| \| --- \| --- \| --- \| --- \| --- \| --- \| --- \| --- \| \|  \|  \|  \|  \|  \|  \|  \|  \|  1. I prefer to receive training or information through the following methods. Please rank your top three preferences from 1 to 3, with 1 being your top preference.   Handouts  In-person training  Online at your own pace  Online virtual instructor  Short training video  Other. Please provide written response about your preferred method to receive training or information   \|  \| \| --- \|  1. Please select the statement that best describes your current workplace.   I work at a store that is part of a regional or national chain.  I work at a store that is part of a local chain (2 stores or more in one town or city).  I work at a store that is an independent store (1 location only).  I am not sure.   1. What types of activities do you perform currently at your workplace? Choose all that apply.   Sales  Cashier  Animal care  Stocking  Supervision of one or more employees  Cleaning of animal housing (e.g. pens, cages, food bowls, water bowls)  Other. If you select Other, please provide a written answer.   \|  \| \| --- \|   32. How long have you been employed at your current workplace?  0 to >6 months  6 months to >2 years  2 to >5 years  <5 years  33. Would you like to be entered for a chance to win a $25 gift card? Your contact information will only be used to contact you if you win the gift card and will not be used for any other purpose.  Yes  No  If Yes please provide:  First Name: _______________________________________________________________  Last Name: _______________________________________________________________  Email Address: ____________________________________________________________ |
| --- | --- | --- | --- | --- | --- | --- | --- | --- | --- | --- | --- | --- | --- | --- | --- | --- | --- | --- | --- | --- | --- | --- | --- | --- | --- | --- | --- | --- | --- | --- | --- | --- | --- | --- | --- | --- | --- | --- | --- | --- | --- | --- | --- | --- | --- | --- | --- | --- | --- | --- | --- | --- | --- | --- | --- | --- | --- | --- | --- | --- | --- | --- | --- | --- |
